# MicroPort CRM considerations on Vega pacing lead performance

**DOI:** 10.1093/europace/euaf060

**Published:** 2025-03-21

**Authors:** Andrea Vincon

**Affiliations:** MicroPort CRM Srl, Via Crescentino SNC, Saluggia 13040, Italy

Website: https://www.microport.com

We read with great interest the article by Özkartal *et al*.,^1^ which describes their single-centre experience with active fixation pacing leads. The authors report a high rate of complications associated with MicroPort’s Vega™ leads compared with other leads (Solia S™, Biotronik; Ingevity™, Boston-Scientific) and the rates published in MicroPort’s Product Performance Reports.

To accurately assess the performance of Vega™ leads, it is crucial to consider the experience gained from multicentre, international, controlled clinical investigations^2–5^ involving these pacing leads. From April 2018 to June 2023, 346 patients were implanted with 541 Vega™ leads (329 atrial and 212 ventricular) across 46 sites in 11 countries. The rate of Vega™ leads complications requiring intervention was 2.6% (1.9% from dislodgments and 0.7% from perforation\pericardial effusions) with a median follow-up of 22 months. These rates are consistent with existing literature.

Furthermore, the pooled multicentre data eliminate any bias that may arise from a single center^6,7^ and the practices of a limited number of healthcare providers.

Regarding the Product Performance Reports, MicroPort adheres to international standards, such as ISO 5841-2 (reporting of clinical performance of populations of pulse generators or leads). It is therefore justified and expected that the complication rates differ from those reported in the article, as they are based on different analysis methods. The authors noted that 45% of complications occurred within 30 days of implantation, while ISO 5841-2 states that acute lead complications must be reported separately in performance reports and not included in lead survival probability. This distinction is critical because acute complications may not be directly attributable to lead design but can result from various factors, including patient-specific anatomy, clinical conditions, implant techniques, and the experience of the implanters.

Additionally, more than 70% of the complications attributed to Vega™ in the article are linked to any pacing threshold increase > 2.0 V, even when capture and safety margins were maintained. However, ISO 5841-2 requires consideration of only significant increases where a 2:1 safety margin can no longer be achieved. Notably, the authors did not report most of the events for vigilance and, therefore, for inclusion in performance reports.

We acknowledge the work of Özkartal *et al*. and include it in our literature reviews. However, the data reported in the article should be evaluated in the context of a retrospective, monocentric cohort analysis. Their results are not in line with global data from larger populations that support the performance of Vega™ in line with the relevant literature and competitors’ leads.
